# Wearable Sensors Integrated with Internet of Things for Advancing eHealth Care

**DOI:** 10.3390/s18061851

**Published:** 2018-06-06

**Authors:** Jose-Luis Bayo-Monton, Antonio Martinez-Millana, Weisi Han, Carlos Fernandez-Llatas, Yan Sun, Vicente Traver

**Affiliations:** 1 Instituto Universitario de Investigación de Aplicaciones de las Tecnologías de la Información y de las Comunicaciones Avanzadas (ITACA), Universitat Politècnica de València, Camino de Vera S/N, Valencia 46022, Spain; anmarmil@itaca.upv.es (A.M.-M.); cfllatas@itaca.upv.es (C.F.-L.); vtraver@itaca.upv.es (V.T.); 2 School of Electronic Engineering and Computer Science, Queen Mary University of London, London E1 4NS, UK; w.han@se13.qmul.ac.uk (W.H.); yan.sun@qmul.ac.uk (Y.S.); 3 Unidad Mixta de Reingeniería de Procesos Sociosanitarios (eRPSS), Instituto de Investigación Sanitaria del Hospital Universitario y Politecnico La Fe, Bulevar Sur S/N, Valencia 46026, Spain

**Keywords:** eHealth, wearable, monitoring, services, integration, IoT, Telemedicine

## Abstract

Health and sociological indicators alert that life expectancy is increasing, hence so are the years that patients have to live with chronic diseases and co-morbidities. With the advancement in ICT, new tools and paradigms are been explored to provide effective and efficient health care. Telemedicine and health sensors stand as indispensable tools for promoting patient engagement, self-management of diseases and assist doctors to remotely follow up patients. In this paper, we evaluate a rapid prototyping solution for information merging based on five health sensors and two low-cost ubiquitous computing components: Arduino and Raspberry Pi. Our study, which is entirely described with the purpose of reproducibility, aimed to evaluate the extent to which portable technologies are capable of integrating wearable sensors by comparing two deployment scenarios: Raspberry Pi 3 and Personal Computer. The integration is implemented using a choreography engine to transmit data from sensors to a display unit using web services and a simple communication protocol with two modes of data retrieval. Performance of the two set-ups is compared by means of the latency in the wearable data transmission and data loss. PC has a delay of 0.051 ± 0.0035 s (max = 0.2504 s), whereas the Raspberry Pi yields a delay of 0.0175 ± 0.149 s (max = 0.294 s) for N = 300. Our analysis confirms that portable devices (p<<0.01) are suitable to support the transmission and analysis of biometric signals into scalable telemedicine systems.

## 1. Introduction

Internet of Things (IoT) is a framework in which sensors, devices and actuators can be managed in an ubiquitous and distributed way [1]. The health sector is not outside of the IoT revolution and there already exist multiple applications and services for improving health care quality [2].

Within this context, telemedicine is an ideal scenario for the expansion and improvement of health IoT technologies [3]. Remote monitoring using accessible and easy-to-use sensors are the avant-garde of the application of these type of technologies [4].

Distributed systems for remote monitoring have been presented elsewhere [5,6] describing two main types of architectures. On the one hand, the first type of system allows storing biometric, behavior and context variables from commercial sensors into devices (mobile phones, tablets and computers) thereafter to generate comprehensive reports to support health-related decision making [7]. On the other hand, the second type of systems automatically forwards the acquired data (without storing it) with Bluetooth or WiFi wireless transmissions [8,9].

To date, one of the largest systems for remote monitoring was deployed and piloted in the *Whole System Demonstrator Programme* (WSD) [10]. This program was promoted by the National Health System of the United Kingdom to stimulate the adoption of telecare. The main purpose was to provide more than 6000 patients with tools to manage their chronic conditions with a tight supervision of clinical staff (up to 238 physicians) through the use of sensors for monitoring physiological signals integrated into a complex communication system. The WSD consisted of three deployments with different technological choices, but the architecture was the same: a base unit to visualize data and questionnaires and peripheral health monitoring devices. Each deployment site used different protocols for allocating sensors: a pulse-oximeter, a glucometer, weighing scales, etc. Data were transmitted from the base unit to a monitoring centre via a secure server Internet connection. These sensors were capable of monitoring very important variables such as blood glucose, body weight, blood oxygenation, pulse and blood pressure, among others, However, these were not integrated with the base unit and mobile devices in every case. The interim results report pilot study was able to show some improvements, but the final report concluded that the intervention group was not benefiting from the use of remote care [11].

Authors of WSD suggest that the impact of remote care interventions are dependent on the architecture and the performance of the system, as other authors confirmed recently [12]. There are qualitative and quantitative tools to measure the user response to a telemedicine system [13]; however, more details about the technical implementation and the technical assessment should be reported to put the results into a context of significance. Clinical outcomes may be distorted by transmission errors, data duplication and missing data due to timeouts.

The Personal Connected Health Alliance (PCHA) (http://www.pchalliance.org/continua/) has gathered more than 200 manufacturers of health sensors and software companies to boost inter-operable eHealth devices and build fully integrated solutions. PCHA was established as a non-profit organization to promote the adoption of medical devices (hardware or software) standards as a way to build complex solutions based on the IoT paradigm. The International Standard Organization (ISO) and the Institute of Electrical and Electronic Engineers (IEEE) launched the 11,073 Communication Standard compendium which describes the behavior, information exchange, nomenclature and connection rules for the health and wellness devices to be integrated into different operational scenarios: from Body Area Networks to location-distributed systems.

However, the complexity of this standard has limited the widespread adoption in the wearable and medical device ecosystem [14]. PCHA certified products are often more expensive than the same product without the communication standard, and, moreover, the adoption of it into commercial sensors is testimonial [15].

Connecting health sensors into the IoT paradigm could be an easy and fast way to deploy complex telemedicine intervention, as these do not need implementing complex connection rules and deep nomenclatures and they put a special focus on the simplicity, interoperability and traceability as the basement for the integration of sensors into a health management system. Health Level 7 Association (HL7) has recently launched Fast Health Interoperability Resources (FHIR) protocol [16], a lite version of HL7 Control Protocol and the Reference Information model aiming to attract developers to build efficient inter-operable solutions [17]. Bluetooth Low Energy (BLE) defines a special profile for Health and Fitness devices, but it has shown several implementation constraints that may reduce BLE performance in a real scenario, in comparison with the theoretically expected [18,19].

Considering the contributions from BLE and FHIR, main shortcomings for prototyping new eHealth solutions under the IoT paradigm are the unnecessary overload of data exchange and difficulties to build demonstration scenarios [20]. At the sensor–device communication level (for instance, electrocardiography sensor to a mobile device), there are a huge amount of headers and data descriptors which are only useful for high communication layers. Although messages should be controlled by standard quality metrics and procedures, adding unnecessary data to wireless physical interfaces may cause more problems than what they actually tend to solve. Moreover, when moving towards a real user case in which patients and health professionals exchange data, there are needs of deploying a Graphical User Interface (GUI), for instance a webpage running on a specific webserver (or even a mobile application), which adds hurdles to the potential and strengths of the interconnection of distributed systems.

In this paper, we present and evaluate an embedded distributed system with a custom lite protocol for the connection of cheap health devices based on prototyping eHealth solutions (Arduino, Raspberry Pi, and biosignals kit). All components are interconnected using the process choreography paradigm [21,22]. To evaluate the extent to which portable devices can be compared with fix systems, the overall deployment has been configured into two different deployment scenarios: (1) Desktop Computer with Windows 10 Operating System; and (2) Raspberry Pi with Windows 10 Core IoT Operating System. The same functionalities for requesting and retrieving data from health sensors and hosting an HTLM5 webpage were embedded into the two systems to compare a key performance indicator: the time delay between acquiring and displaying the bio-signal. Our experiments confirm the expected hypothesis, that is: portable components have an increased latency in the communications, but this latency is negligible. Portable devices are suitable to support the transmission and analysis of biometric signals into scalable telemedicine systems. Our conclusion is that portable computing fosters new opportunities to expand the use of wearables in health care research. Strengths of the proposed system are the open specification of the protocol, the open-communications method using standard communication structures (based on XML) and choreography, and the direct connection to open-source hardware components. These results make it possible to enhance the system for other domains, such as Ambient Assisted Living (AAL) and smart-home sensors.

## 2. Material and Methods

In this section, we describe the materials and methodology we used to test the two deployment scenarios for a remote health management system. We first describe the hardware to sense biometric signals using the eHealth Sensors kit and Arduino. Second, we describe the integration paradigm based on service choreography. Third, we describe the communication protocol. Finally, we describe the experimental setup.

### 2.1. eHealth Sensors Kit

Arduino is an open-specification platform based on an ATmega328P micro-controller with the minimum capacity to execute simple programs (sketches). Arduino provides an easy but effective hardware to connect and use many electronic components with a wide variety of applications [23,24]. The official website of Arduino [25] has a comprehensive collection of information, downloads, tutorials and examples about how to use the platform.

Arduino provides an excellent platform to test and prototype solutions by adding supplemental modules (Bluetooth, WiFi boards, LEDs, and servo-motors) and other hardware. In this research, we have set up the eHealth Biometric Sensor Platform created by Libelium [26]. This kit allows users to acquire a set of physiological signals such as ECG, EMG, breathing rate, surface temperature, GSR, blood glucose and SpO2 (Table 1). eHealth kit has been used in wearable sensors research [27,28].

The Libelium kit is not a certified medical device. We used this platform as a virtual medical device because it implements the same physical interfaces and monitoring circuits as certified and commercial sensors do. Moreover, it prevents us from developing manufacturer protocols to retrieve raw physiological signals to test our principal hypothesis.

**Figure 1:**
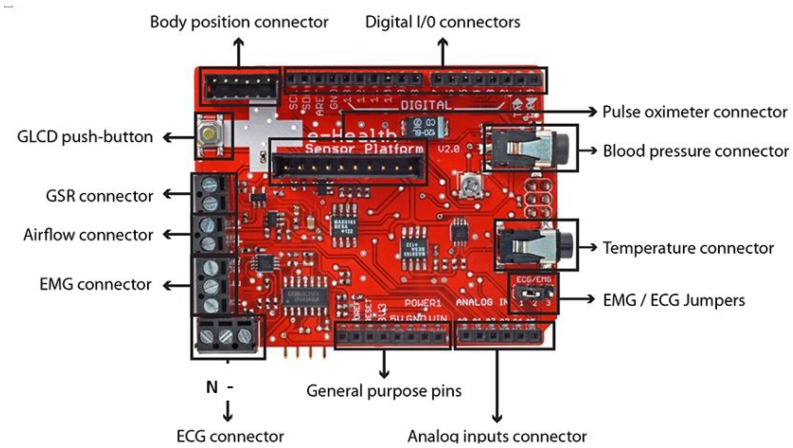
eHealth shield input/output pins [26].

The whole sensing information detection part for biometric information relies on the Arduino eHealth Sensor Kit (Figure 1).

These sensors can be used as diagnostic tools in clinical settings and also used to remotely monitor the state of a patient in real time. Some of the sensors which use the electro-physiological techniques (e.g., ECG, EMG, and GSR) require a time series to make a sense of them. All biometric sensors should be integrated on one Arduino board to collect comprehensive biometric information at the same time.

### 2.2. Choreography Integration

One of the objectives to improve health care services is to provide reliable remote access to the data retrieved by the sensors of the eHealth kit. A Service Oriented Architecture (SOA) involves the use of weakly coupled services to support such processes in a high inter-operable way. In an SOA environment, network resources are available through services which can be accessed through standard methods. Masking such resources with services allows accessing them without the needs of knowing how they were implemented internally.

The software enabling communication between the sensors and the displaying interfaces was implemented using a choreography engine [12], a semantic engine capable of connecting registered services and functions. The Choreographer dispatches messages among the modules using a specific eXtensible Markup Language (XML) message protocol called eXtensible MeSsaGe (XMSG) [21]. XMSG is based on the Foundation for Intelligent Physical Agents (FIPA) recommendations [29] and Simple Object Access Protocol (SOAP) [30] headers to route and characterize messages. The XMSG protocol allows broad-/multi-cast, as well as Peer to Peer (P2P) message calls, using custom symbols in the destination address. XMSG can be serialized and transmitted over any transport protocol such as REST and HTTP.

**Figure 2:**
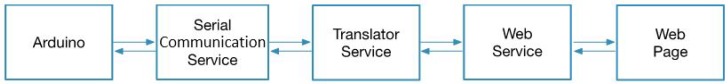
Choreography integration.

**Figure 3:**
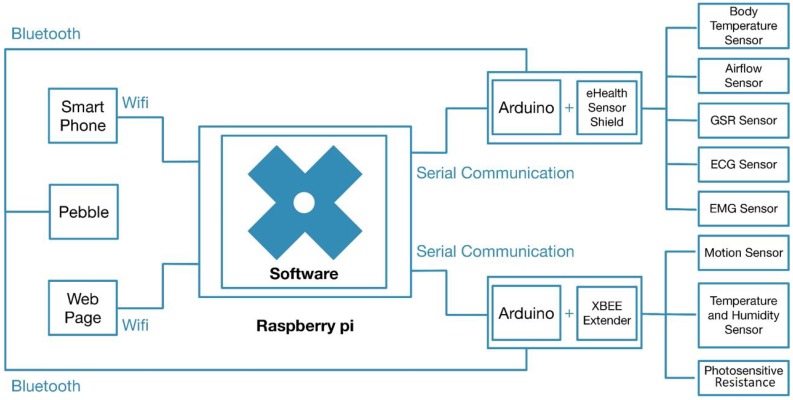
System architecture including health wearable sensors.

Services are designed to fit into three categories: serial communication, data translation and web-client. The client part is defined as a web service to deliver an interactive interface in a web browser. Serial communication service establishes the connection with the Arduino hardware to read data from wearable sensors. The translator service verifies data packets and translates information according to a predefined communication protocol. After extracting data from the packets, a new message is generated and sent to the client service to be displayed in web interface. These services also allow controlling the sensors (turn on/off, change sampling frequency, etc.). Figure 2 draws the information flow between the hardware and the interactive interface. Figure 3 shows a schema of the physical connection of the components, enhancing the information flow of Figure 2. In this schema, we show how the wearable sensors are connected to the Arduino + eHealth shield and how this component enables the communication to the Choreographer software (blue cross) in the Raspberry and other applications in the Pebble smart watch. In this paper, we have analyzed the flow of information through the serial communication port to the Choreographer, and then, from the Choreographer to the webpage by using WiFi interface. The webpage is a front-end which can be executed in a browser on any computer/tablet.

### 2.3. Communication Protocol

Once the sensors have been connected to the eHealth shield, the most important issue is to define a communication protocol to ensure correct data transmission. As illustrated in Figure 3, information should be handled between the webpage and the Arduino board through a distributed system (Sensors + Arduino in Location A and the Webpage in Location B). This type of information involves sensor data (type of measurement, value, units, time stamp, etc.) and the control commands that manage the sensors. Besides, error detection mechanism should also be added since the messages may be corrupted during the transmission.

Two operational modes are defined for operating with sensors. This first is the Active mode, through which sensors can automatically collect and send measurements to the software at every time interval according to their individual settings. Thus, data from all sensors can be displayed in real-time. The second is the passive mode, which implies that users can send different commands from the webpage to a specific sensor to request its data or change acquisition parameters. Users should be capable of interacting and operating with sensors: to request the current value of a sensor, to stop data retrieval, to set the time interval, etc. The structure of the commands is described in Table 2 and Table 3. Therefore, the protocol should consider the following descriptors: **Kit Type**: Type of the sensor kit used for extensions to other type of sensing environments such as smart homes. **Destination**: Name of the sensor which identifies the destination of the command. **Command**: Different commands which can be used to ask for the sensor data or change some default settings. **Parameter**: This field corresponds to the Command field for changing some settings (e.g. transmit the parameter of the time interval in active mode). **Checksum**: The checksum field is the 16 bit sum of all bytes of the command packet. It can be used to detect command error which might be introduced during the transmission. **Sensor Type**: Name of the sensor which sends data or response. **Response To**: This field indicates which command the sensor is responding to, so the communication can be asynchronous. **Data**: This is the data part measured by the sensors or the answer of the command. **Unit**: This field corresponds to the Data field and it is the unit of the data.

The format of the sensor data packet and response packet is shown in Table 3.

### 2.4. Portable Embedded System

Raspberry Pi was introduced in 2012 [31] and has been extensively used in home monitoring environments [32,33]. It integrates a computer board with support for many input and output peripherals through standard interfaces (Serial, Bluetooth and Wi-Fi) and allows the installation of several operative systems. Dimensions of the device are 85.60 mm × 53.98 mm × 17 mm, with a weight of approximately 45 grams. The device is cased and mounted inside a plastic box with a 7 inch touchscreen and powered with a battery. Raspberry Pi 3 is a platform suitable for embedding high level applications which allow the interaction with different devices and users in a wide range of applications. In this study, we used the Raspberry Pi 3 with the operating system from Microsoft Windows 10 IoT Core [34]. Windows 10 IoT Core is an optimized version of Windows 10 for small portable ARM and ×86/×64 devices. One of the aims of this paper is to evaluate the extent to which portable systems are ready to substitute computers in the way they connect and process information coming from several eHealth sensors. To this end, we will evaluate the Raspberry Pi 3 in comparison to a desktop computer.

### 2.5. Design of Experiments

The proposed system must provide a record track of the executed services, their results, time stamp and other audit information. A track component should be in charge of recording the trace of all the activities that take place during the performance of the system. The records must be standardized (or even normalized), understandable and ready to be parsed and mined. Therefore, this component will record all interaction events among the modules and components.

The experiments assessed the communication delay between distributed physical components among service request and service response for all integrated sensors. The main key performance indicator is the latency, which is defined as the difference of time between the start of the transmission of the first message and the end of the correct reception of the last message [18]. The latency wasmeasured and compared in two environments using the same choreography software:

- Raspberry Pi 3 device with a Windows 10 IoT Core Operating System. Processor ARMv8 at 1.2 GHz and 1 GB of RAM.
- Desktop computer with a Windows 10 Operating System. Processor Dual Core at 2.6 GHz and 4 GB of RAM.

Due to the skewed distribution of the latency parameters, a Wilcoxon signed-rank test at 95% C.I. was used to assess the independence of intra and inter schema differences. Significance was assumed for *p* < 0.05. Statistical and graphical analysis was done using Matlab 2016R version using Academic License.

## 3. Results

The choreography engine uses SOA and implements a Windows Presentation Foundation GUI [35] application with several services declared and running in the background. Each service represents a specific functionality and different services interact with each other by passing messages.

### 3.1. Choreographer Functions to Manage eHealth Sensors

**Figure 4:**
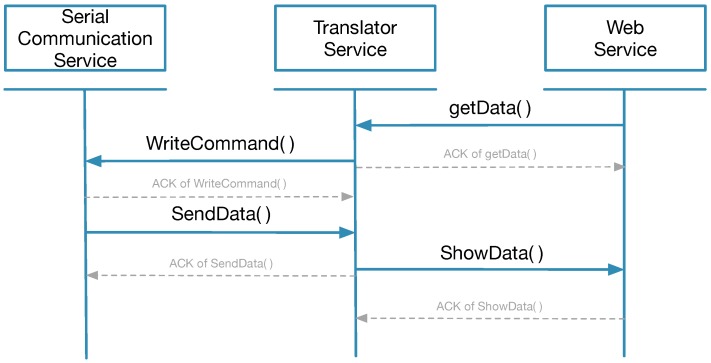
Passive mode.

**Figure 5:**
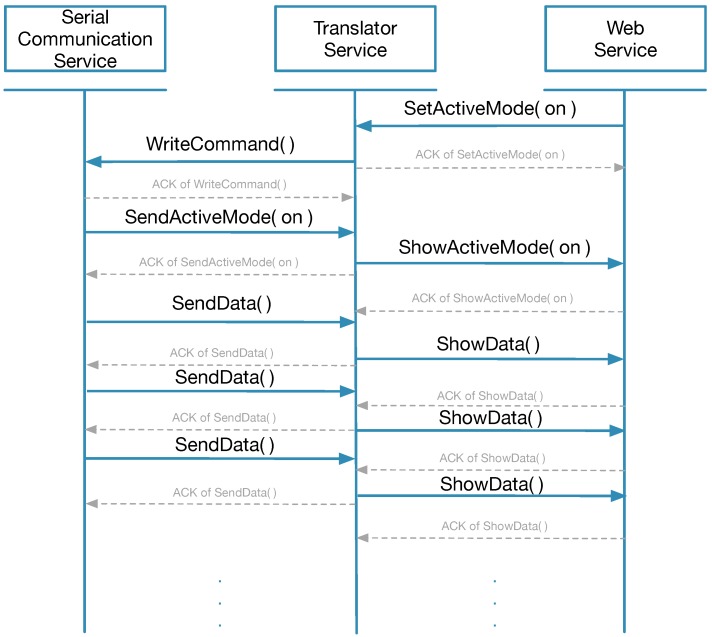
Active mode.

The services for accessing eHealth sensors are managed by the choreography engine (Table 4). As shown in Section 2.2, the Arduino board communicates with the serial communication service, and, analogously, users interacts with the web dashboard to request data from sensors. The translator service performs as a middleware by connecting the web service and serial communication service while data are exchanged between entities. Figure 4 and Figure 5 show the sequence diagram of the three services.

Illustrated by the sequential diagram in Figure 5 and Table 5, the translator service is one of the most crucial parts of the overall architecture since it is the only service which implements the communication protocol. Each sensor has one translator and different methods are used to parse the information coming from the serial communication service or encapsulate various commands received from web service into command packets.

Each sensor has an individual webpage to control and display biometric signals. Therefore, for passive mode, once a user taps a button on the webpage to request data or set up operational modes, the web service sends a command to the corresponding translator. The translator service identifies the intent of the request and translates it into the predefined format, adding header and checksum to the command, and then sends the restructured new message to serial communication service. When the Arduino board receives the message from serial communication service, it retrieves *Destination* and *Command* information to effectively execute the command (e.g., read ECG).

After executing the command, the Arduino board generates a response packet for answering the request. The serial communication service transmits the packet to all translator services and each translator has to verify and parse it if it is a valid message. Translator service is able to distinguish which request this packet answers by means of *Response To* field. The *Data* part and *Units* part are then extracted and transmitted to the target web service by referring to *Sensor Type* part of the packet. Finally, data are shown on the webpage using a time series chart.

To illustrate the operation in detail, we can take body temperature data request as an example. When the user clicks the button on the webpage to request body temperature, the Body Temperature Web Service calls *getData* method in Body Temperature Translator Service and the translator starts to construct a command packet. It sets the *Kit Type* field as *EHEALTH*, *Destination* field as *BODYTEMPERATURE*, *Command* field as *GETDATA*, no *Parameter* and uses delimiter *‘|’* to concatenate each individual field. After calculating the checksum of the whole command, it is appended as the tail of the packet and the resulting command packet stands as *“EHEALTH|BODYTEMPERATURE| GETDATA||2657”*.

Similarly, when the Arduino board finishes reading the command, it creates a data packet and sends it to the serial communication service. The data packet could look like *“EHEALTH|BODYTEMPERATURE|GETDATA |36.5|C|3052”*. Later, it will be transmitted to Body Temperature Translator Service, where the checksum of this packet will be calculated again and compared with the original one. If there is a match, the packet is validated.

For the active mode, the web service should send a *setActiveMode* command with parameter *on*. Once the active mode has been configured, the Arduino board will send the specific sensor data at a predefined rate. This behavior will not stop until another *setActiveMode off* command is triggered. When it comes to sensors such as the ECG, Airflow and GSR, the active mode can be extremely important. These continuous data are collected and utilized to generate a biometric signal for real-time monitoring.

### 3.2. Track Component

**Figure 6:**
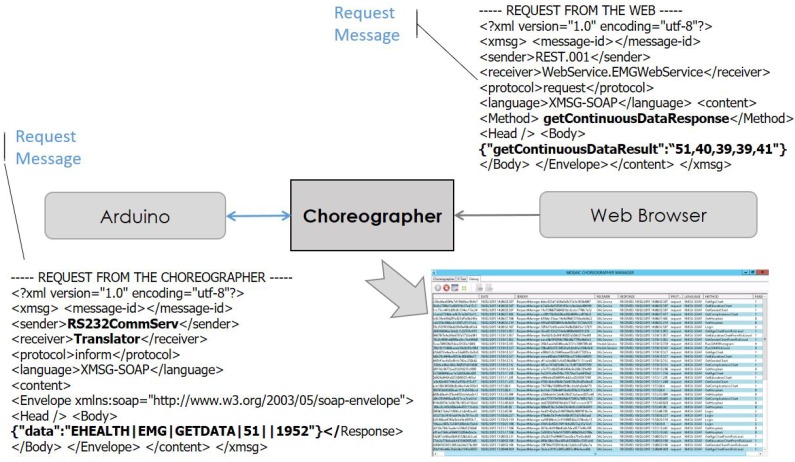
Choreographer track service.

The Choreographer included a service to keep track of all the exchanged messages across components. As the limitations on the time resolution deserved special attention and open a brand new study field, all the interactions on the webpage were recorded in a special format and placed in a basic Comma-Separated Values file (to make easy the access for the information). A file named *choreo_track* was automatically generated upon first launch of the system (see Figure 6). A main class controlled the interaction events during a session and tracked them in that file. Each interaction event was written in a line with the following format:
*<Timestamp>, <sender>, <receiver>, <message>*

- Time stamp: dd/mm/yyyy hh:mm:ss.sssss.
- Sender: The module who triggered or controlled the action (see Figure 2).
- Destination: The module who was receiving the service request.
- Method: To indicate whether it was a request or an inform method.
- Message: The data exchanged, for instance the packets described in previous sections.

### 3.3. Raspberry Pi for Hosting and Serving a Webpage

**Figure 7:**
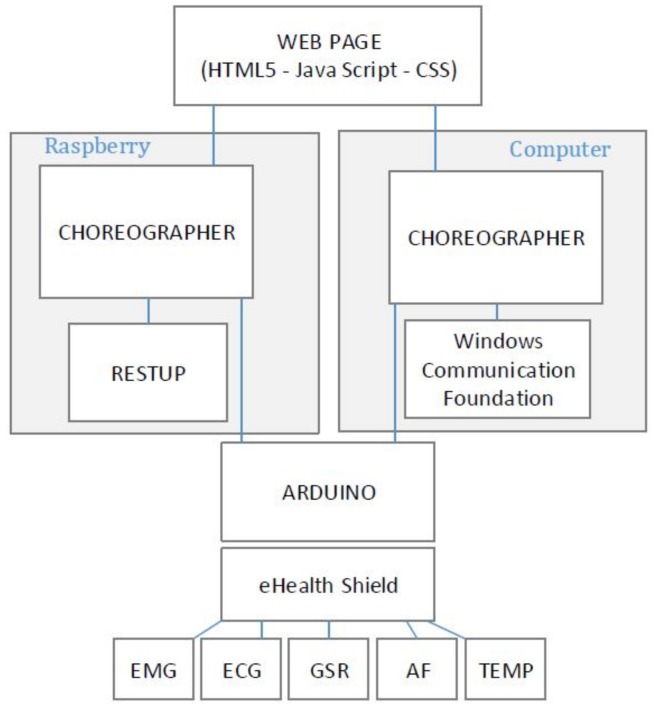
Services for hosting and serving the webpage. The schema shows how the Choreographer is connected to the sensors through the Arduino module and the needed libraries to self host and serve the webpage, which is based on HTML5 + Java Script + CSS.

Web service representation for both scenarios are based on RESTful (Representational State Transfer) service. A service in the Choreographer is in charge of serving a webpage based on JavaScript and HTML5. The implementation is based on the .NET v4.5 framework for webservers and implementation of RESTful services. As Figure 7 shows, in the case of the computer implementation, libraries are directly used from Windows Communication Foundation (WCF), whereas, for the Raspberry, the use of RESTUP libraries was needed [36]. RESTUP maps web service libraries from WCF to be compatible with Windows 10 IoT Core operating system. Other studies have shown good results by implementing this architecture based on Apache Server [37].

### 3.4. Deployment

**Figure 8:**
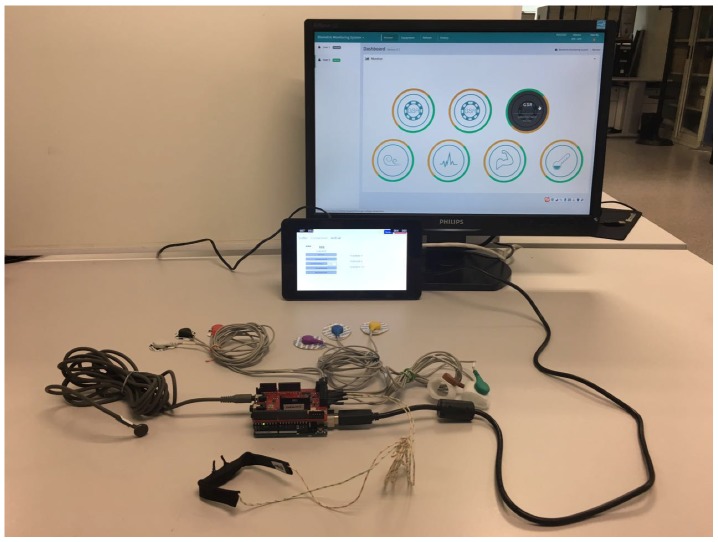
Sensor deployment.

Figure 8 illustrates the final system. The picture shows the first term the Arduino Uno mounted with the eHealth shield and the health sensors kit. Different sensors should be connected to the eHealth shield. The EMG sensor and the ECG cannot work simultaneously since a jumper should be switched in the board to use one adaptation circuit or another. The Arduino is connected through a USB interface to the Raspberry Pi or the PC, in which an instance of the Choreographer is installed.

**Figure 9:**
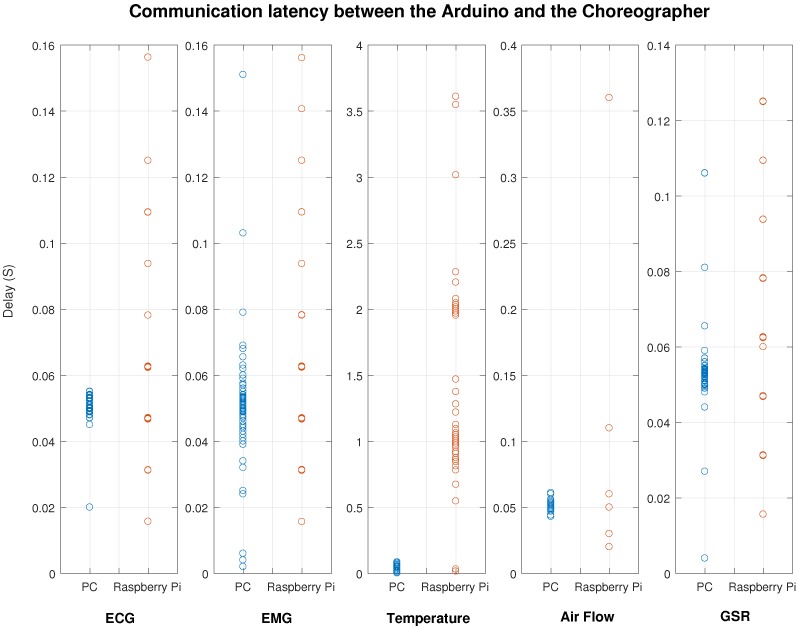
Comparison of the delay in the communications for the system deployed on a desktop computer and a Raspberry Pi for the segment between the Arduino and the Choreographer for the active communication mode.

**Figure 10:**
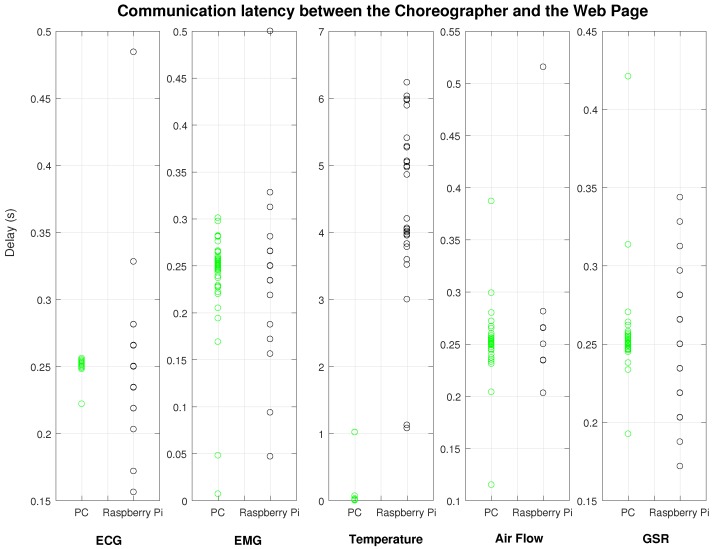
Comparison of the delay in the communications for the system deployed on a desktop computer and a Raspberry Pi for the segment between the Choreographer and the webpage with the active communication mode.

The computer screen shows the welcome page of the web application, which shows access to analyze data from the five sensors (the three buttons are for the three variables of the GSR sensor). After clicking on each button, the user can access individual pages for each sensor where different operations can be executed. For sensors such as ECG, EMG, GSR and Airflow sensor, single datum seems meaningless, thus users can start active mode to monitor and graph real-time sensor data. Besides, it is also feasible for users to set a time interval to retrieve past values from the sensors. Once these values are prepared, a chart would be plotted for each different set of data. The chart is real-time updated (see time delays in Figure 9 and Figure 10), and it is refreshed every 250 ms. For body temperature sensor, apart from the above functions, users can access current body temperature data by clicking *GETDATA* button.

The Raspberry Pi (below the screen) shows the control Graphical User Interface of the Choreographer. The Choreographer was created similar to a tab in our window, containing graphical services which can be used to manage the five sensors in the system. By doing so, users can interact with the sensors without the needs of accessing the webpage.

### 3.5. Experiments

Sensors that support the active mode (ECG, EMG, Airflow and GSR) have a default sampling rate set to 20 Hz (20 Samples per second); this means that the interval between two samples is 50 ms for both PC and Raspberry Pi. Besides, the webpage is set to request data every 250 ms. The experiment shows that the PC deployment generated less latency in the communication segment between the Arduino and the Choreographer (Figure 9). Our measurements correspond to the theoretical sample period, which is 50 ms. Nonetheless, we do not see this behavior in the Raspberry Pi: The mean value of the time delay is around 50 ms, but the standard deviation is greater than the standard deviation of the PC. These points are distributed sparsely but showed the pattern of an arithmetic series. The gap between two corresponding points is approximately 10 ms to 20 ms.

The communication of the active mode between Choreographer and webpage (Figure 10) shows a similar situation. The PC shows a good result with all the measurements concentrated around 250 ms. We see a different pattern in the Raspberry PI with all the measurements with a fixed interval (mean value is 250 ms and the gap is about 10 ms to 20 ms).

We compared the communication segment between the Arduino and the Choreographer for the two deployments (Raspberry and PC). This segment is composed of the Serial Communication Service and the Translator service. Results show that Raspberry Pi has a statistically significant increased delay (p<<0.01) for all the five sensors, whereas, for the segment between the Choreographer and the web service, all sensors except ECG showed a not statistically significant increased delay (for ECG p<<0.01, and p>0.5 for the rest).

Figure 9 and Figure 10 show the cumulative scatter plots for the delays tracked with the tracker component (Figure 6). Even though the results are scattered, it is notorious that the delay experimented for the Raspberry Pi shows a clear pattern (especially for ECG, EMG and GSR in Figure 10). This pattern may be caused by the internal delay of the Arduino for acquiring measurements (10 ms–20 ms), and the reason of not having it on the PC may depend of the communication stack, which is bigger in the PC. In this case, the Choreographer can fill the memory stack with more measurements which will be delivered faster to the web interface.

Another finding involves the precision of time intervals. The PC shows a bigger reliability by means of a lower standard deviation and a lower range. As an example, for the EMG communication between the Arduino and the Choreographer (Figure 9), the PC has a delay of 0.051±0.0035 s and a range of 0.2504 s (N = 300), whereas the Raspberry has a delay of 0.0175±0.149 s and a range of 0.294 s (N = 300). Therefore, the Choreographer achieves a higher reliability for acquiring measurements if deployed in a PC.

## 4. Discussion

In this paper, we present an integration of two innovative paradigms: Health Sensors and IoT focused on simple deployments. These two streams, which are widely accepted by the scientific community, are seen as the future of how information and communication technologies can make health care system sustainable. Moreover, the connection of these two paradigms with new ubiquitous computing and artificial intelligence models can promote the crucial step-forward for the adoption and spread use of health sensors for the management of chronic conditions.

In this context, forecasts on the increment of the population over 60 years old in developed countries and the pandemic dimension some diseases are reaching deserves special attention. Health care systems are not prepared to sustain these numbers and there is increased demand for better and personalized care services. This is the main reason that pushes us to propose cheap and scalable solutions that allow users to plug and play them without the need of understanding complex standards or programming frameworks.

One of the flagship projects on the evaluation of remote care presented in Section 1 (Whole System Demonstrator Programme) has demonstrated favorable results on the management of patients with chronic conditions, but stating that the technology is not yet ready for scaling-up [11]. The system proposed and evaluated in this study is based on a Service Oriented Architecture with a central component (Choreographer). The Choreographer works as a message dispatcher that allows connecting different modules (health sensors, webpages, etc.). The simplicity of the Choreographer prevents us from using another type of complex integration solutions like Enterprise Serial Bus (ESB), which provides a gain on the performance of its execution and fault tolerance [20].

The Choreographer relies on existing libraries and communication stacks of Microsoft .NET v4.5 framework, a key element for ensuring its reutilization and the compatibility with legacy systems in hospitals and health care infrastructures. The Choreographer implements a protocol based on a custom formatting message exchange language, XMGS. XMSG is based on SOAP and not REST to achieve a minimum level of standardization of information exchange across the system. Nevertheless, as reported in Section 3, REST stands as a proper methodology for describing the interfaces of the system by simply serializing XSMG. The proposed architecture can be adapted to a RESTful architecture with JSON messages, but due to the complexity of the distributed system, the number of sensors and client applications, it is needed to ensure a minimum set of information into the exchanged messages.

The eHealth sensing platform has been built on purpose using the Arduino Libellium kit, a low-cost solution that allows the integration of a wide range of physiological sensors. Even though these sensors are for prototyping, they are useful to work with on the design, development and evaluation of possible solutions to record, store and transmit biometric signals (ECG, EMG, Airflow, etc.). This technology was chosen over other commercial health sensors because of the cost as well as the integration simplicity: Arduino provides serial communication over wired and wireless physical interfaces, whereas the majority of sensors only provide wired communication, and, most of the time, the protocols to retrieve measurements are not available for third parties or are based on complex standards as the ISO/IEEE 11073 [14].

The proposed system is capable of hosting and serving a website as a regular web service. This is an extremely important feature that allows certifying the system as plug-and-play. With the proposed architecture of communications, health sensors may be configured to be subscribed to a specific website to perform a real-time broadcasting of the measurements, at the same time that these measurements may be stored in cloud services or analyzed by complex algorithms.

The deployment of this system is feasible on either a PC or a Raspberry Pi. According to our experiments (Figure 9 and Figure 10), there is a statistically significant increased delay on the Raspberry Pi with respect the PC, which is understandable considering the unbalanced set of computational resources. However, for some specific cases, this delay is not relevant for the medical and monitoring purpose; (in the case of ECG, latency difference is below 0.030 s).

The significantly increased delay of Raspberry Pi may be related to the processor technical features, which has fewer capabilities than the personal Computer processor (ARMv8 @1.2 GHz and 1 GB RAM versus Dual Core @2.6 GHz and 4 GB RAM). Future work should consider experimental verification of the causes of the delay. Our results show that the latency experimented from side-to-side (meaning from sensors to the webpage) has not a big difference (even though significant), and the delay introduced by the Raspberry Pi is assumable. The low costs and requirements of a Raspberry Pi are not comparable with the high costs and requirements of a personal computer. Therefore, our experiments suggest that the new architectures for monitoring bio-signals could rely on the implementation of networks based on the Raspberry Pi without compromising the latency.

## 5. Conclusions

In this manuscript, we present and evaluate a scalable system based on five wearable sensors that allow the plug-and-play deployment on different use scenarios (on Raspberry and desktop computer). Raspberry pi yields a significant increased delay with respect to the same implementation in a personal computer. However, the measured delay is negligible and acceptable in real-time remote monitoring. Implementation of health web sensor node as a part of the Internet of Things using a Raspberry Pi has benefits with respect the use of a desktop computer, which paves the way to the implementation of new portable systems for remote management of chronic conditions. Future work will pursue on this line, finding out the percent of missing packets and the throughput in terms of other KPIs, such as the error rate, memory use, power consumption and influence of the network load (4 G/5 G). Moreover, future research should implement large amounts of traffic (several users and longer periods) to reflect the performance differences in commercial environments.

## Figures and Tables

**Table 1:** eHealth sensors.

Sensor Name	Description
Temperature Sensor	Body temperature depends upon the place in the body in which the measurement is made, and the time of day and the level of activity of the subject. Different parts of the body have different temperatures.
	**Discrete data**
Airflow Sensor	Respiratory rate is a broad indicator of major physiological instability. The sensor measures the breathing flow of a person in the up airways (nose). Airflow sensor can also provide an early warning of hypoxemia and apnea.
	**Continuous data**
Galvanic Skin Response Sensor (GSR)	It can be used to measure the electrical conductance of the skin, which varies with its moisture level. Skin conductance is used as an indication of psychological or physiological arousal. GSR measures the electrical conductance between 2 points, and is essentially a type of ohmmeter.
	**Continuous data**
Electrocardiography Sensor (ECG)	A diagnostic tool that is routinely used to assess the electrical and muscular functions of the heart. ECG has grown to be one of the most commonly used medical tests in modern medicine. Some diseases have no modifications on ECG waveform.
	**Continuous data**
Electromyogram Sensor (EMG)	It can be used as a diagnostic tool to evaluate and record the electrical activity produced by skeletal muscles by measuring the electrical activity of muscles at rest and during contraction. EMG is used for identifying neuromuscular diseases, assessing low-back pain, kinesiology, and disorders of motor control.
	**Continuous data**

**Table 2:** Text-based Command format.

Header		Data		Tail
Kit Type	Destination	Command	Parameter	Checksum
		Field Delimiter	‘|’	

**Table 3:** Sensor data and response format.

Header		Data			Tail
Kit Type	Sensor Type	Response To	Data	Unit	Checksum
		Field Delimiter	‘|’		

**Table 4:** Classification and list of services in the Serial Communication and the RS232 Communication components.

Serial Communication Service	RS232 Communication Service
Translator Services	Airflow Translator
	Body Temperature Translator
	ECG Translator
	EMG Translator
	GSR Conductance Translator
	GSR Resistance Translator
	GSR Conductance Voltage Translator
Web Services	Airflow Web Service
	Body Temperature Web Service
	ECG Web Service
	EMG Web Service
	GSR Conductance Web Service
	GSR Resistance Web Service
	GSR Conductance Voltage Web Service

**Table 5:** Methods implemented for the information exchange between the communication services and their description.

Web Service → Serial Communication Service	
getData()	Request for the sensor data one real-time.
SetActiveMode(onOff)	If the parameter is *on*, start active mode of the target sensor and request for the continuous data. If the parameter is *off*, stop active mode.
ChecksumCalculate(command)	Calculate the checksum of the full command and append it at the end of the packet.
**Serial Communication Service** → **Service**	
sendData(figure, unit)	Send the sensor data and unit
sendActiveMode(figure)	Send the response of the SetActiveMode command
ChecksumCheck(old_cks, new_cks)	Calculate the checksum of the received data
packet and compared it with the one in the packet.	

## Abbreviations

The following abbreviations are used in this manuscript:

AF	Air Flow
ECG	ElectroCardioGraphy
EMG	ElectroMioGraphy
GSR	Galvanic Skin Response
HL7	Health Level 7
PCHA	Personal Connected Health Alliance
SOA	Service Oriented Architecture
